# Presurgical Characteristics and Seizure Outcomes in Patients With Focal Cortical Dysplasia Type I, II, and III: A Single‐Center Study

**DOI:** 10.1002/brb3.71053

**Published:** 2025-11-27

**Authors:** Lei Jin, Dixuan Zhi, Yanfeng Yang, Yihe Wang, Di Lu, Kaishan Wang, Yongzhi Shan

**Affiliations:** ^1^ Department of Neurosurgery, Xuanwu Hospital Capital Medical University Beijing China; ^2^ China International Neuroscience Institute (CHINA‐INI) Beijing China

**Keywords:** drug‐resistant epilepsy, epilepsy surgery, focal cortical dysplasia, surgical outcomes

## Abstract

**Aims**: This study aims to investigate the presurgical characteristics of different types of focal cortical dysplasia (FCD) and analyze the impact of these factors on postoperative seizure outcomes.

**Methods**: A retrospective analysis was conducted on 77 patients with histopathologically confirmed FCD, categorized into types I, II, and III. Clinical data and preoperative examinations were compared among the three groups, with significant differences analyzed using multinomial logistic regression. Cox regression was used to assess the impact of presurgical factors on postoperative seizure outcomes.

**Results**: Significant differences were found between FCD types I, II, and III in lesion location (*p* = 0.001) and the use of stereoelectroencephalography (SEEG) (*p* = 0.017). FCD type II lesions were more often extratemporal and unilateral (*p* < 0.05), while type I patients were more likely to require SEEG (*p* < 0.05). A trend toward a lower risk of seizure recurrence was observed in patients over 21 years old at the time of surgery (HR = 0.376, *p* = 0.045), although this association was not statistically significant after Benjamini−Hochberg false discovery rate (FDR) adjustment (FDR‐adjusted *p* = 0.495).

**Conclusion**: FCD type II lesions are predominantly extratemporal and unilateral, while SEEG is more commonly needed in type I cases. Additionally, surgical outcomes in adults are not inferior to those in children, indicating that epilepsy surgery is an effective treatment for both age groups.

## Graphical abstract

This retrospective cohort study demonstrated that FCD type II lesions are predominantly extratemporal and unilateral, while SEEG is more commonly needed in type I cases. Additionally, surgical outcomes in adults are similar to those in children, indicating that epilepsy surgery is an effective treatment for both age groups.

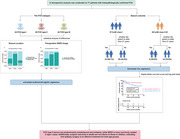

## 1 Introduction

Focal cortical dysplasia (FCD), first described by Taylor et al. in the 1970s (Taylor et al. 1971), is a common cause of drug‐resistant epilepsy in both children and adults (Blumcke et al. 2017). It is estimated that approximately 18.7%–40% of pediatric epilepsy patients and 19.8% of adult epilepsy patients have FCD (Blumcke et al. 2017; Liu et al. 2022; Kun et al. 2020). Among individuals with FCD, 87% will develop epilepsy, and about 75% of those will become drug‐resistant (Cohen et al. 2023). A large retrospective cohort study (Cohen et al. 2023) found that failure of even a single antiseizure medication (ASM) is associated with a high risk of drug resistance, prompting suggestions to redefine drug resistance in FCD as failure of one ASM. This perspective carries significant implications for the timing of surgical interventions.

Epilepsy surgery has been demonstrated to be an effective treatment for FCD, with seizure freedom rates reported between 55.2% and 88% (Willard et al. 2022; Cohen et al. 2022; Jayalakshmi et al. 2022; Xue et al. 2016). Limited evidence suggests that postoperative seizure outcomes are influenced by histopathological subtypes. Patients with FCD type II typically have favorable surgical outcomes, with seizure freedom rates ranging from 85% to 92% (Usui et al. 2024; Chassoux et al. 2022). In contrast, surgical outcomes for FCD type I are less favorable, with seizure freedom rates reported between 42.4% and 71% (Okumura et al. 2024; Splitkova et al. 2025). Outcomes for FCD type III largely depend on the nature of the associated lesion (Najm et al. 2022). To date, numerous studies (Willard et al. 2022; Xue et al. 2016; Chassoux et al. 2022; Wagstyl et al. 2022) have explored predictors of postoperative seizure freedom in FCD. Among these, the most consistent predictor is the completeness of lesion resection (Willard et al. 2022; Fauser et al. 2015). Other factors, such as histological subtype, the use of intracranial Electroencephalography (EEG), lesion location, and age at surgery, remain controversial regarding their predictive value (Willard et al. 2022; Xue et al. 2016; Chassoux et al. 2022; Wagstyl et al. 2022).

In 2022, the International League Against Epilepsy (ILAE) updated the histopathological classification of FCD based on its 2011 classification. However, the classifications of FCD types I, II, and III remained largely unchanged (Najm et al. 2022; Blümcke et al. 2011). FCD type I lesions tend to be more diffuse than type II, often requiring more extensive resection to achieve seizure freedom. Accurate presurgical identification of FCD subtypes, particularly between types I and II, may help tailor surgical strategies and improve outcomes (Najm et al. 2022; Krsek et al. 2009; Janca et al. 2023).

This retrospective study analyzed 77 patients at our epilepsy center who had a postoperative histopathological diagnosis of FCD and had failed treatment with at least one ASM prior to surgery. All patients had a minimum postoperative follow‐up of 1 year. The aim of this study was to evaluate basic clinical data and presurgical assessments to facilitate differentiation among FCD types I, II, and III, thereby contributing to improved surgical outcomes. In addition, we further examined whether the predictive factors for surgical outcomes, previously debated in the literature, hold prognostic value in our cohort of FCD patients.

## 2 Method

### 2.1 Patients and Data Collection

We retrospectively analyzed 118 patients with drug‐resistant epilepsy who underwent epilepsy surgery at the Department of Neurosurgery, Xuanwu Hospital, Capital Medical University, China, between May 2019 and December 2023, and were histopathologically diagnosed with FCD.

Inclusion criteria were as follows: (1) histopathological confirmation of FCD type I, II, or III according to the 2022 ILAE classification criteria (Najm et al. 2022); (2) availability of at least 12 months of postoperative follow‐up data; and (3) failure to achieve seizure freedom despite treatment with at least one ASM.

Exclusion criteria included: (1) histopathological diagnosis of mild malformations of cortical development (mMCDs), mild malformations of cortical development with oligodendroglial hyperplasia in epilepsy (MOGHE), or no definite FCD on histopathology according to the 2022 ILAE classification (Najm et al. 2022); (2) unavailability of preoperative magnetic resonance imaging (MRI) or incomplete basic clinical data.

**FIGURE 1:**
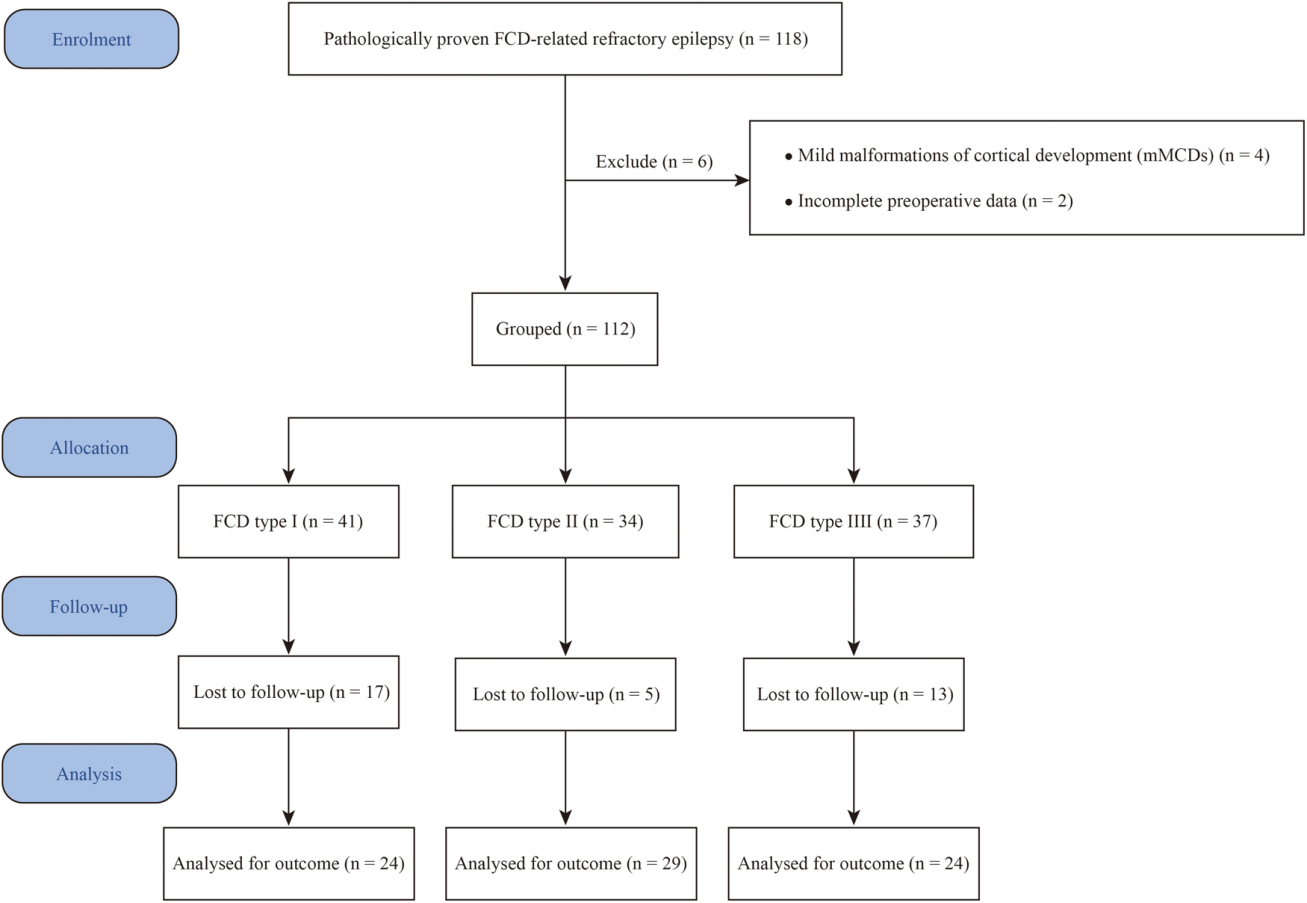
Flow diagram of patient selection. A total of 118 patients with histologically confirmed FCD were evaluated. After excluding 4 with mMCDs, 2 with missing preoperative data, and 35 lost to follow‐up (17 FCD I, 5 FCD II, 13 FCD III), 77 patients were included in the final analysis (24 FCD I, 29 FCD II, 24 FCD III).

After applying the above inclusion and exclusion criteria, four patients were excluded due to a histopathological diagnosis of mMCDs, and two were excluded due to missing preoperative data. In addition, 35 patients who had undergone surgery but were lost to postoperative follow‐up were excluded from the outcome analysis, despite having available histopathological diagnoses. As a result, a total of 77 patients were included in the final analysis (Figure 1).

The retrospective analysis included the following variables: sex, age at epilepsy surgery, seizure duration, history of febrile seizures, preoperative use of ASMs, whether ASMs were adjusted, lesion location, and histopathological findings. This study was approved by the Ethics Committee of the Xuanwu Hospital, Capital Medical University, and written informed consent was obtained from all patients.

### 2.2 Preoperative Evaluation

All patients underwent a comprehensive presurgical evaluation, including high‐resolution MRI and video‐electroencephalography (vEEG) based on the international 10–20 system. When conventional MRI failed to clearly identify the lesion, additional imaging modalities such as 18F‐fluorodeoxyglucose positron emission tomography (FDG‐PET), magnetoencephalography (MEG), or stereoelectroencephalography (SEEG) were utilized to aid in lesion localization. SEEG was performed based on two principal clinical indications: (1) when noninvasive evaluations failed to clearly localize the epileptogenic zone, such as in cases with discordant or inconclusive findings across modalities including MRI, FDG‐PET, MEG, and video EEG; and (2) when noninvasive studies suggested that the epileptogenic zone was located within or in close proximity to eloquent cortex, thereby necessitating precise delineation to guide surgical planning (Isnard et al. 2018). All presurgical data were reviewed and discussed by a dedicated multidisciplinary team, comprising specialists in neurosurgery, neurology, and pediatrics. An appropriate surgical strategy was then determined and carried out by the neurosurgical team.

### 2.3 MRI Study

All preoperative MRI data were acquired at Xuanwu Hospital, Capital Medical University, using a 3T scanner (GE Healthcare, Waukesha, WI) with a 64‐channel head‐neck combined coil. The imaging protocol adhered to the HARNESS‐MRI recommendations (Bernasconi et al. 2019) and comprised high‐resolution three‐dimensional (3D) T1‐weighted imaging, high‐resolution 3D fluid‐attenuated inversion recovery (FLAIR), and high in‐plane resolution two‐dimensional (2D) coronal T2‐weighted imaging oriented perpendicular to the long axis of the hippocampus.

All MRI images were evaluated by a dedicated multidisciplinary team in conjunction with other presurgical data (PET, MEG, SEEG), without prior knowledge of the histopathological diagnosis or precise lesion location. MRI positivity was defined as the presence of any of the following visually detectable abnormalities: (1) cortical thickening; (2) transmantle sign; (3) gray‐white matter junction blurring; (4) abnormal gyral or sulcal morphology; (5) focal white matter volume loss; (6) signal hyperintensity on FLAIR sequences; (7) hippocampal atrophy and/or signal abnormalities.

### 2.4 Surgical Approach

At our center, all lesions were treated using extended lesionectomy, defined as resection of the visible lesion along with an additional safety margin of approximately 5–10 mm. This margin was determined on a case‐by‐case basis by a multidisciplinary team. Specifically, the extent of the epileptogenic zone was identified preoperatively using MRI and FDG‐PET, and, when necessary, MEG or SEEG. Intraoperatively, electrocorticography was used to detect abnormal discharges, and electrical stimulation mapping was performed to localize eloquent cortex. When safely feasible, resection was extended by 5–10 mm beyond the defined epileptogenic zone. In cases where the lesion overlapped with eloquent cortex, a maximal safe resection was performed based on intraoperative electrocorticography findings.

### 2.5 Neuropathological Analysis

All resected specimens were analyzed and classified by a single experienced neuropathologist at the Department of Neuropathology, Xuanwu Hospital, Capital Medical University, according to the ILAE guidelines on FCD proposed by Najm et al. in 2022 (Najm et al. 2022). This study included only patients diagnosed with FCD types I, II, or III. Given the substantial pathological heterogeneity among FCD III subtypes, we reported the available subclassification of FCD III cases for reference. For FCD I and II, consistent subclassification was not reported, as in a subset of patients, the histopathological distinction between subtypes was difficult; therefore, only the main categories could be reliably assigned.

### 2.6 Postoperative Follow‐Up

Postoperative follow‐up was conducted using a standardized protocol, with the first assessment at 3 months after surgery, the second at 6 months, the third at 1 year, and annually thereafter. All patients included in this study were followed up for at least 1 year. Follow‐up methods included telephone interviews, outpatient visits, or inpatient evaluations. Seizure outcomes were assessed according to the 2017 ILAE classification system (Scheffer et al. 2017), which is defined as follows: Class 1 = completely seizure‐free; Class 2 = only auras, no other seizures; Class 3 = 1–3 seizure days per year, regardless of auras; Class 4 = more than 4 seizure days per year, with up to a 50% reduction from baseline, regardless of auras; Class 5 = less than 50% reduction in seizure days compared to baseline, regardless of auras; Class 6 = more than 100% increase in seizure days compared to baseline, regardless of auras.

Our clinical team collected postoperative outcome data, which was subsequently reviewed and consolidated by a single clinician. Adjustments to ASM regimens were made during follow‐up when clinically indicated. Specifically, ASM was adjusted in patients who experienced unprovoked seizures after surgery. For seizure‐free patients with normal follow‐up EEG and more than 3 years after surgery, gradual tapering of ASM was considered. The seizure outcomes reported in this study were based on the results of the most recent follow‐up.

### 2.7 Statistical Analysis

Statistical analysis was performed using IBM SPSS Statistics 27.0 for Windows (Chicago, IL, USA) and R software (version 4.5.1). A two‐sided *p*‐value < 0.05 after Benjamini−Hochberg false discovery rate (FDR) adjustment was considered statistically significant. Adjusted *p*‐values were reported alongside unadjusted ones (Tables 1, 4, and 5).

**TABLE 1:** Clinical data and MRI findings in patients by FCD subgroups.

Variables		FCD I (*n* = 24)	FCD II (*n* = 29)	FCD III (*n* = 24)	H/χ^2^/F	*p* value	Adjusted *p* value
Seizure duration, years		8.250 (4.000, 13.000)	6.000 (2.500, 16.000)	9.000 (3.475, 19.750)	0.622	0.733	0.733
Age at epilepsy surgery, years		22.625 ± 10.265	17.966 ± 11.870	23.875 ± 13.231	1.864	0.162	0.297
FU, years		4.645 (3.240, 5.390)	3.820 (2.660, 4.710)	4.530 (3.543, 5.333)	4.337	0.114	0.297
No. of preop ASMs		2.000 (2.000, 3.000)	2.000 (2.000, 4.000)	2.000 (1.000, 2.750)	3.881	0.144	0.297
ASMs adjustment					0.866	0.649	0.714
	Adjustment	15 (62.5%)	17 (58.6%)	17 (70.8%)			
	Not adjustment	9 (37.5%)	12 (41.4%)	7 (29.2%)			
Sex					3.089	0.213	0.307
	Male	11 (45.8%)	17 (58.6%)	17 (70.8%)			
	Female	13 (54.2%)	12 (41.4%)	7 (29.2%)			
Seizure outcome at last FU					2.409	0.300	0.367
	ILAE class 1	15 (62.5%)	23 (79.3%)	19 (79.2%)			
	ILAE class 2–6	9 (37.5%)	6 (20.7%)	5 (20.8%)			
MRI visibility					4.375	0.112	0.297
	Positive	15 (62.5%)	23 (79.3%)	21 (87.5%)			
	Negative	9 (37.5%)	6 (20.7%)	3 (12.5%)			
Lesion location					23.242^*^	**< 0.001**	**0.001**
	Temporal lobe	12 (50%)	5 (17.2%)	15 (62.5%)			
	Extratemporal lobe	8 (33.3%)	23 (79.3%)	4 (16.7%)			
	Multiple lobe	4 (16.7%)	1 (3.4%)	5 (20.8%)			
Preop SEEG					11.511	**0.003**	**0.017**
	Yes	12 (50.0%)	6 (20.7%)	2 (8.3%)			
	No	12 (50.0%)	23 (79.3%)	22 (91.7%)			
Febrile seizures					3.026^*^	0.223	0.307
	Yes	4 (16.7%)	3 (10.3%)	7 (29.2%)			
	No	20 (83.3%)	26 (89.7%)	17 (70.8%)			

*Note*: Continuous variables are presented as mean ± standard deviation (SD) or median with interquartile range (IQR), depending on distribution. Categorical variables are presented as counts and percentages. Boldface type indicates statistical significance. Adjusted *p*‐values are based on FDR correction to account for multiple comparisons.

Abbreviations: FCD, focal cortical dysplasia; FU, follow‐up; No. of preop ASMs, number of preoperative antiseizure medications; preop, preoperative.

^*^Indicates *p*‐values calculated using Fisher's exact test.

#### 2.7.1 Descriptive Statistics

Continuous variables are presented as mean ± standard deviation (SD) or median with interquartile range (IQR), depending on distribution. Categorical variables are presented as counts and percentages.

#### 2.7.2 Group Comparisons

The Shapiro–Wilk test was used to assess normality, and Levene's test was used to evaluate homogeneity of variance. Comparisons of presurgical clinical features across FCD subtypes were performed using one‐way ANOVA or Kruskal–Wallis tests for continuous variables, and chi‐square or Fisher's exact tests for categorical variables, as appropriate. Similarly, comparisons between patients with favorable versus unfavorable postoperative seizure outcomes were conducted using independent‐sample *t* tests, Mann–Whitney U tests, chi‐square, or Fisher's exact tests as appropriate.

#### 2.7.3 Predictive Modeling

To assess the association between FCD subtype (FCD types I, II, and III) and individual predictive factors, a multinomial logistic regression analysis was performed. Variables included in the model were those that showed statistically significant differences among FCD subgroups in univariate comparisons, specifically lesion location and use of SEEG prior to surgery.

Univariate Cox proportional hazards models were used to identify predictors of time to seizure recurrence. The proportional hazards assumption was verified using Schoenfeld residuals (“cox.zph” function in R), and no violations were detected. Variables with *p* < 0.05 (prior to FDR adjustment) in univariate analyses were retained for further discussion, upon which Kaplan–Meier survival curves and log‐rank tests were subsequently applied to evaluate seizure outcomes and explore the prognostic value of age >21 years at the time of surgery. FDR correction was also applied to *p*‐values in univariate Cox analyses. No multivariable modeling was performed due to the limited sample size and event rates.

## 3 Results

### 3.1 Clinical Data and Preoperative Predictors of Histology

The 77 included patients were categorized into three groups based on the histopathological diagnosis of the resected specimens: 24 patients (31.17%) were classified as FCD type I, 29 patients (37.66%) as FCD type II, and 24 patients (31.17%) as FCD type III. Among the 24 FCD III cases, subclassification per the 2022 ILAE criteria was available: 16 were FCD IIIa and 8 were FCD IIId. No FCD IIIb or IIIc cases were identified in this cohort. The mean (± SD) age at epilepsy surgery was 22.63 (± 10.27) years for FCD type I, 17.97 (± 11.87) years for FCD type II, and 23.88 (± 13.23) years for FCD type III. The baseline clinical characteristics of the three groups are summarized in Table 1. Analysis showed no statistically significant differences among the three groups in terms of sex (*p* = 0.213), age at epilepsy surgery (*p* = 0.162), seizure duration (*p* = 0.733), number of preoperative ASMs (*p* = 0.144), whether ASMs were adjusted (*p* = 0.649), follow‐up duration (*p* = 0.114), seizure outcomes (*p* = 0.300), MRI findings (*p* = 0.112), or history of febrile seizures (*p* = 0.223). However, significant differences were observed in lesion location (FDR‐adjusted *p* = 0.001) and use of SEEG prior to surgery (FDR‐adjusted *p* = 0.017).

Subsequently, univariate multinomial logistic regression analyses were conducted for lesion location and preoperative use of SEEG. Interestingly, we found that, compared to extratemporal unilobar lesions, temporal lobe lesions were associated with a significantly increased likelihood of being classified as FCD type I (vs. FCD type II), with an odds ratio (OR) of 6.9 (*p* = 0.004). Similarly, multilobar lesions were associated with an even higher risk of FCD type I (vs. FCD type II), with an OR of 11.5 (*p* = 0.040). In addition, temporal lobe lesions (vs. extratemporal unilobar lesions) were associated with an increased risk of FCD type III (vs. FCD type II), with an OR of 17.25 (*p* < 0.001), while multilobar lesions (vs. extratemporal unilobar lesions) had an OR of 28.75 (*p* = 0.006), both statistically significant (see Table 2). Furthermore, patients who underwent SEEG prior to surgery had significantly lower odds of being classified as FCD type II (vs. FCD type I), with an OR of 0.261 (*p* = 0.029), and FCD type III (vs. FCD type I), with an OR of 0.091 (*p* = 0.004), indicating a significant association (see Table 3). Additionally, in patients with FCD type II, the majority of lesions were located in the frontal lobe unilaterally (18/29, 62.07%).

**TABLE 2:** Multinomial logistic regression analysis of predictive factor of the FCD II.

Factor	Temporal lobe versus extratemporal lobe			Multiple lobe versus extratemporal lobe		
	OR	95% CI	*p* value	OR	95% CI	*p* value
FCD I	6.90	1.848–25.763	**0.004**	11.50	1.114–118.707	**0.040**
FCD II	contract	—	—	—	—	—
FCD III	17.25	3.979–74.792	**< 0.001**	28.75	2.621–315.409	**0.006**

*Note*: Boldface type indicates statistical significance.

**TABLE 3:** Multinomial logistic regression analysis of predictive factor of the FCD I.

Factor	Preoperative SEEG versus no preoperative SEEG		
	OR	95% CI	*p* value
FCD I	contract	—	—
FCD II	0.261	0.078–0.869	**0.029**
FCD III	0.091	0.017–0.475	**0.004**

*Note*: Boldface type indicates statistical significance.

These results suggest that, in FCD type II patients, lesions are predominantly extratemporal and typically confined to a single (especially frontal) lobe, whereas patients with FCD type I more frequently require SEEG prior to surgery for precise epileptogenic zone localization.

### 3.2 MRI Findings

Among patients with FCD type I, 15 cases (62.5%) were MRI‐positive; in FCD type II, 23 cases (79.3%) were MRI‐positive; and in FCD type III, 21 cases (87.5%) were MRI‐positive. Although the differences in MRI positivity rates among the three groups were not statistically significant (*p* = 0.112), there was a trend toward a lower MRI detection rate in patients with FCD type I.

Among patients who were seizure‐free postoperatively (ILAE class 1), 45 cases (78.9%) were MRI‐positive. In patients with postoperative seizures (ILAE classes 2–6), 14 cases (70%) were MRI‐positive. The difference between the two groups was not statistically significant (Yates's corrected chi‐square test, *p* = 0.613).

### 3.3 Preoperative Predictors of Long‐Term Seizure Outcome

The 77 included patients were divided into two groups based on postoperative seizure outcomes: 57 patients (74.03%) were classified as ILAE class 1, and 20 patients (25.97%) were classified as ILAE classes 2–6. The median follow‐up time was 4.37 years (range from 1.2 to 5.98 years). The average age at the time of surgery was 21.26 years (SD = 11.99). Detailed clinical information for both groups is presented in Table 4. Analysis revealed no statistically significant differences between the two groups in terms of seizure duration (*p* = 0.834), follow‐up duration (*p* = 0.732), number of preoperative ASMs (*p* = 0.976), whether ASMs were adjusted (*p* = 0.883), sex (*p* = 0.717), pathological subtype (*p* = 0.3), MRI findings (*p* = 0.613), lesion location (*p* = 0.365), use of SEEG prior to surgery (*p* = 0.285), or history of febrile seizures (*p* = 0.927). Although older age at surgery appeared to be significantly associated with a favorable seizure outcome in univariate analysis (*p* = 0.044), the association did not remain significant after adjusting for multiple comparisons (FDR‐adjusted *p* = 0.484).

**TABLE 4:** Clinical data and MRI findings in patients by seizure outcome subgroups.

Variables		ILAE class 1 (*n* = 57)	ILAE class 2–6 (*n* = 20)	χ^2^/U	*p* value	Adjusted *p* value
Seizure duration, years		8.000 (3.650, 16.000)	7.000 (3.000, 13.000)	552.000	0.834	0.976
FU, years		4.350 (2.800, 4.920)	4.545 (3.170, 4.885)	540.500	0.732	0.976
No. of preop ASMs		2.000 (2.000, 3.000)	2.000 (1.250, 3.750)	567.500	0.976	0.976
ASMs adjustment				0.022	0.883	0.976
	Adjustment	36 (63.2%)	13 (65.0%)			
	Not adjustment	21 (36.8%)	7 (35.0%)			
Sex				0.132	0.717	0.976
	Male	34 (59.6%)	11 (55.0%)			
	Female	23 (40.4%)	9 (45.0%)			
Age at epilepsy surgery, years				4.047	**0.044**	0.484
	≤ 21	25 (43.9%)	14 (70.0%)			
	> 21	32 (56.1%)	6 (30.0%)			
Histology				2.409	0.3	0.976
	FCD I	15 (26.3%)	9 (45.0%)			
	FCD II	23 (40.4%)	6 (30.0%)			
	FCD III	19 (33.3%)	5 (25.0%)			
MRI visibility				0.256^*^	0.613	0.976
	Positive	45 (78.9%)	14 (70.0%)			
	Negative	12 (21.1%)	6 (30.0%)			
Lesion location				2.014	0.365	0.976
	Temporal lobe	26 (45.6%)	6 (30.0%)			
	Extratemporal lobe	25 (43.9%)	10 (50.0%)			
	Multiple lobe	6 (10.5%)	4 (20.0%)			
Preop SEEG				1.145	0.285	0.976
	Yes	13 (22.8%)	7 (35.0%)			
	No	44 (77.2%)	13 (65.0%)			
Febrile seizures				0.008^*^	0.927	0.976
	Yes	11 (19.3%)	3 (15.0%)			
	No	46 (80.7%)	17 (85.0%)			

*Note*: Continuous variables are presented as mean ± standard deviation (SD) or median with interquartile range (IQR), depending on distribution. Categorical variables are presented as counts and percentages. Boldface type indicates statistical significance. Adjusted *p*‐values are based on FDR correction to account for multiple comparisons.

Abbreviations: FCD, focal cortical dysplasia; FU, follow‐up; No. of preop ASMs, number of preoperative antiseizure medications; preop, preoperative.

^*^Indicates *p*‐values calculated using Yates's corrected chi‐square test.

Next, univariate Cox regression analysis was performed to identify predictive factors significantly associated with postoperative seizure freedom (ILAE class 1). Because the mean age at surgery in our cohort was 21.26 years and the age distribution was approximately normal, we used 21 years as a data‐driven cutoff for dichotomization. Using this threshold, we initially found that patients older than 21 years had a significantly higher probability of postoperative seizure freedom (*p* = 0.045), with a hazard ratio (HR) of 0.376 compared to those aged 21 years or younger, as detailed in Table 5. However, this association did not remain statistically significant after FDR correction (FDR‐adjusted *p* = 0.495). The proportional hazards assumption was tested and was not violated (global Schoenfeld test *p* = 0.767; Table 5).

**TABLE 5:** Univariate Cox regression analysis of predictive factors of the seizure outcome for FCD patients.

Variable		HR	95% CI	*p* value	Adjusted *p* value	Schoenfeld test *p*
Sex		1.277	0.529−3.087	0.587	0.717	0.671
Seizure duration		0.992	0.942−1.044	0.748	0.750	0.471
Age at epilepsy surgery (cutoff ≤ 21)		0.376	0.144−0.979	**0.045**	0.495	0.723
No. of preop ASMs		1.178	0.759−1.828	0.466	0.675	0.826
Preop SEEG		0.562	0.224−1.409	0.219	0.574	0.492
Febrile seizures		0.819	0.24−2.798	0.750	0.750	0.854
MRI visibility		1.399	0.538−3.642	0.491	0.675	0.585
Lesion location						0.115
	Temporal lobe	0.358	0.101−1.271	0.112	0.674	
	Extratemporal lobe	0.583	0.183−1.861	0.362	0.664	
	Multiple lobe	contract	—	—		
Histology						0.483
	FCD I	contract	—	—		
	FCD II	0.530	0.188−1.49	0.229	0.574	
	FCD III	0.534	0.179−1.594	0.261	0.574	

*Note*: Boldface type indicates statistical significance. Adjusted *p*‐values are based on FDR correction to account for multiple comparisons.

Abbreviations: FCD, focal cortical dysplasia; No. of preop ASMs, number of preoperative antiseizure medications; preop, preoperative.

**FIGURE 2:**
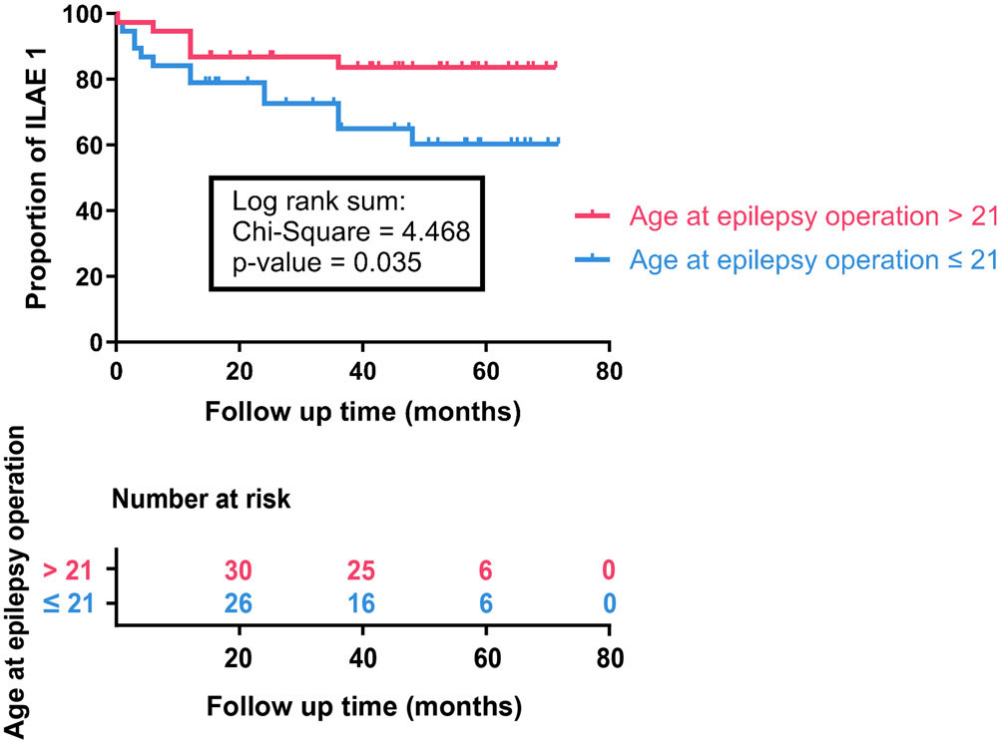
Kaplan–Meier survival curves comparing postoperative ILAE class 1 status between different age groups at the time of epilepsy surgery. Kaplan–Meier curves show the proportion of patients achieving ILAE class 1 (complete seizure freedom) over the follow‐up period (0–71.73 months) for two age groups: age at epilepsy surgery > 21 years (red line) and age at epilepsy surgery ≤ 21 years (blue line). Numbers at risk for each group are provided below the x‐axis. Statistical analysis using the log‐rank test demonstrated a significant difference between the two groups (chi‐square = 4.468, *p* = 0.035). Patients who underwent epilepsy surgery at an age > 21 years were more likely to achieve and maintain ILAE class 1 compared to those who underwent surgery at an age ≤ 21 years.

Finally, Kaplan−Meier survival curves and Log‐rank tests showed that patients older than 21 years at the time of epilepsy surgery had a significantly higher likelihood of postoperative seizure freedom (Figure 2).

These results support the idea that older age at the time of surgery does not imply worse postoperative outcomes, and may even suggest a trend toward improved surgical prognosis in older patients.

## 4 Discussion

This study demonstrates significant differences in lesion location and the need for presurgical SEEG to define the extent of the lesion among different pathological subtypes of FCD. Additionally, a potential trend toward improved seizure outcomes in older patients was observed, but the association was not statistically significant after adjusting for multiple comparisons.

FCD is one of the most common lesions found in epilepsy surgery specimens from both children and adults, and is closely associated with severe drug‐resistant focal epilepsy. Complete resection of the dysplastic cortical lesion can lead to a cure through epilepsy surgery (Blumcke et al. 2017; Willard et al. 2022; Chassoux et al. 2022; Salemdawod et al. 2022; Choi et al. 2018). Normally, cortical development spans from the eighth to the 24th week of gestation and occurs in three stages: the proliferation phase of undifferentiated cells, the migration phase of neuroblasts, and the differentiation phase of cells. Disruption of any step in these stages can lead to cortical malformations, which is the underlying cause of this lesion (Gaitanis 2013).

The distribution of FCD is uneven, with a primary concentration in the superior frontal gyrus, frontal pole, and temporal pole (Wagstyl et al. 2022). This study found that FCD type II lesions are primarily located in the extratemporal regions, particularly in the frontal lobe, while FCD type I and type III lesions are more commonly found in the temporal lobe. These findings are consistent with the research by Chassoux (2022) and Wagstyl (2022). Understanding the typical locations of FCD lesions not only aids in preoperative localization of the lesions but also facilitates FCD subtype classification, thereby guiding surgical planning and improving postoperative epilepsy outcomes. For example, the extent of FCD type I lesions is usually more extensive than what is visible on imaging, often requiring a broader resection to achieve seizure freedom (Janca et al. 2023). A recent study has also reported that FCD type II lesions exhibit a more localized area of hyperexcitability both temporally and spatially (Shahabi et al. 2021). This finding further supports the view that FCD type I lesions have a larger extent than FCD type II lesions, necessitating more extensive resection.

There is a significant amount of research related to the imaging characteristics of FCD, particularly on MRI. As a result, some of the imaging features of FCD have become well‐established. MRI characteristics of FCD type I include reduced white matter volume (Okumura et al. 2024; Colombo et al. 2009), blurring of the gray‐white matter junction (Okumura et al. 2024), mild hyperintensity on FLAIR sequences (Colombo et al. 2009), and abnormal gyral morphology (Krsek et al. 2009). MRI features of FCD type II include cortical thickening (Shahabi et al. 2021), FLAIR signal enhancement (Shahabi et al. 2021), blurring of the gray‐white matter junction (Krsek et al. 2009), abnormal gyral morphology (Krsek et al. 2009), and the transmantle sign in FCD type IIb (Mellerio et al. 2012). The imaging characteristics of FCD type III are related to associated lesions, such as hippocampal atrophy, mass lesions, and “popcorn‐like changes” (Wang et al. 2023). In most patients with FCD type I, these abnormalities are typically not visible on MRI, resulting in negative imaging findings (Blümcke et al. 2011; Shahabi et al. 2021). Although our study did not find that the MRI‐negative rate for FCD type I (37.5%) was significantly higher than that for FCD type II (20.7%) or FCD type III (12.5%), there was a trend in that direction. Nevertheless, the proportion of MRI‐negative cases among FCD type I patients in our cohort was lower than generally expected. This may be attributable to our evaluation procedure, in which initial neuroradiology reports were systematically reviewed in a multidisciplinary epilepsy surgery conference that incorporated other presurgical data such as PET and MEG. Although the reviewers were blinded to the final histopathological diagnosis, the integration of multimodal information likely enhanced the detection of subtle abnormalities. At the same time, this may also account for the finding that, in our series, a significantly larger proportion of patients with FCD type I required SEEG implantation for accurate lesion localization prior to surgery compared with those with FCD type II and type III.

Salemdawod et al. (2022) investigated the long‐term seizure freedom outcomes in FCD patients following surgery, and found that seizure freedom results in the first year post‐surgery were comparable to long‐term outcomes, suggesting that the first year after surgery could be a reliable predictor of long‐term seizure freedom. Cohen et al. (2022) proposed that failure of a single ASM is significantly associated with an increased risk of drug resistance (OR = 346) and an earlier onset of pharmacoresistance, and therefore, the definition of drug‐resistant epilepsy in FCD patients may need to be revised to include the failure of just one ASM. Based on these conclusions, the inclusion criteria for this study were set to require at least 1 year of postoperative follow‐up, and patients who had failed to respond to at least one ASM were included.

In our cohort of patients (median follow‐up of 4.37 years), the overall postoperative seizure‐free rate was 74.03%, which is similar to the findings reported by Cohen et al. (median follow‐up of 43.8 months) (Cohen et al. 2022) and Jayalakshmi et al. (follow‐up duration of 2–13 years) (Jayalakshmi et al. 2022), who reported an overall postoperative seizure‐free rate of 72% in their single‐center case series. Additionally, our findings are consistent with a meta‐analysis (Willard et al. 2022) that reviewed 35 studies, including 1353 FCD patients, which reported a 70% postoperative seizure‐free rate.

FCD is prevalent among patients with drug‐resistant epilepsy, which has led to increasing attention from the medical community. To date, numerous studies have been conducted on FCD, particularly focusing on predictive factors related to surgical outcomes, which are valuable for preoperative counseling for patients and their families (Okumura et al. 2024). Okumura et al. (2024) found that postoperative seizure outcomes depend on the underlying pathology and that temporal lobe resection yields better outcomes than extratemporal resection. Xue et al. (2016) suggested that preoperative electrode implantation is a predictor of poorer surgical outcomes. Wagstyl et al. (2022) argued that the location of FCD is a key determinant of postoperative seizure freedom. Spliykova's study (2025) found that patients with FCD type I have poorer postoperative seizure freedom compared to those with FCD type II. A single‐center study from Germany (Salemdawod et al. 2022) identified complete resection of the epileptogenic focus, the number of ASMs used preoperatively, and younger age at surgery as predictors of long‐term postoperative seizure outcomes in FCD type II patients. While these studies identified protective or risk factors associated with FCD surgical outcomes, they also examined other factors. However, these additional factors did not achieve statistical significance. For instance, a meta‐analysis (Willard et al. 2022) indicated that good postoperative outcomes were associated with complete resection of the FCD lesion and temporal lobe involvement, but not with lesion size, intracranial EEG use, or histological subtype of FCD. To our knowledge, the most consistent predictor of surgical outcomes is the complete resection of the epileptogenic focus (Blumcke et al. 2017; Willard et al. 2022; Salemdawod et al. 2022; Choi et al. 2018). This study aimed to validate whether the aforementioned controversial factors have predictive value in our cohort. The results show that, aside from age at surgery, no other factors were significantly associated with seizure outcomes, as detailed in Tables 4 and 5. It is noteworthy that previous studies have reported poorer postoperative outcomes for patients with FCD type I compared with those with FCD type II (Splitkova et al. 2025). In contrast, our cohort did not reveal significant differences in seizure outcomes among FCD subtypes. A plausible explanation is the relatively high rate of SEEG implantation in FCD type I patients in our center, which may have enabled more accurate delineation of the epileptogenic zone and thereby improved surgical outcomes.

A trend toward improved postoperative seizure freedom was observed in patients older than 21 years at the time of surgery. This finding contrasts with the views of Salemdawod et al. (2022), who suggested that younger age at surgery is a predictor of long‐term seizure freedom in FCD type II patients. In a long‐term follow‐up study, Fauser et al. (2015) also noted that age older than 18 years at surgery is a negative predictor of good postoperative outcomes. Although our initial univariate Cox regression analysis suggested that patients older than 21 years at the time of surgery had a significantly lower risk of postoperative seizure recurrence (HR = 0.376, *p* = 0.045), this association did not remain statistically significant after adjustment for multiple comparisons using the FDR method (adjusted *p* = 0.495). The observed trend may have been influenced by cohort characteristics: all patients were treated at a single, high‐volume epilepsy center in China, where the average age at surgery was relatively high (21.26 ± 11.99 years). This may have introduced a degree of selection bias and limited the generalizability of the results to broader populations. Interestingly, there were no significant differences in age distribution across FCD subtypes (*p* = 0.162, Table 1), indicating that the effect of age was not confounded by histopathological subtype. In addition, we selected 21 years as the dichotomization threshold because the mean age at surgery in our cohort was 21.26 years, making this a data‐driven cutoff for statistical comparison. Moreover, the limited sample size may have affected statistical power. Nevertheless, we wish to emphasize that, in our study, adult patients achieved seizure outcomes comparable to those of younger patients, supporting the notion that favorable outcomes can still be achieved when epilepsy surgery is performed in adulthood for patients with FCD. In other words, we support the view that surgery in adulthood can still lead to good seizure freedom. Ramírez‐Molina et al. (2017) and Chern et al. (2010) both believed that the likelihood of benefiting from surgery is similar for both children and adults. Martinez‐Lizana et al. (2018) reviewed 113 FCD patients aged 0–18 years and found that children older than 9 years tend to have more limited surgical resections but generally better long‐term seizure freedom, suggesting that older patients have more precise lesion localization. Krsek, in comparing his own pediatric case series (Krsek et al. 2009) with adult series (Tassi et al. 2002; Widdess‐Walsh et al. 2005), concluded that adults with FCD type I tend to have better seizure prognosis.

This study has several limitations. First, it is a single‐center retrospective study that included only patients with FCD types I, II, and III, which introduces some selection bias. Second, this study primarily focused on basic preoperative clinical information and simple MRI findings. Data on detailed neurophysiological and advanced neuroimaging characteristics were limited. In particular, postoperative MRI was not routinely obtained, as many patients were followed remotely or underwent imaging at outside institutions. As a result, we were unable to assess the extent of resection, and thus could not evaluate the relationship between surgical completeness and postoperative seizure outcomes, despite its well‐established prognostic significance (Willard et al. 2022; Fauser et al. 2015). Third, although lesion location was significantly associated with FCD subtype, some odds ratios—such as that for multilobar lesions (vs. extratemporal unilobar lesions) in FCD III (OR = 28.75, 95% CI = 2.62–315.41)—were extremely large and accompanied by wide confidence intervals, suggesting potential overestimation due to small subgroup sizes. Additionally, the apparent association between temporal lobe lesions and FCD type I may partly reflect the predominance of FCD type II in the frontal lobe, rather than indicating a true preferential localization of FCD type I in the temporal lobe. This distribution pattern may be influenced by the relative imbalance in lobar representation among different FCD subtypes. Therefore, in future studies, we plan to include more relevant clinical data and conduct a more detailed analysis.

## 5 Conclusion

Our study demonstrates that FCD type II lesions are primarily extratemporal, predominantly unilateral, and display a preference for the frontal lobe, whereas patients with type I FCD more frequently require SEEG for precise localization of the epileptogenic zone. Furthermore, our findings indicate that surgical outcomes in adult FCD patients are not inferior to those in pediatric patients, suggesting that adults are also appropriate candidates for epilepsy surgery. These data provide valuable reference points for presurgical classification and prediction of surgical outcomes in FCD.

## Author Contributions

L.J., D.Z., and Y.Y. contributed to the conception and design of the study. Y.W., D.L., and K.W. contributed to data collection. L.J., D.Z., and Y.Y. contributed to the analysis of data. L.J., D.Z., Y.Y., Y.W., D.L., and K.W. contributed to drafting the text, preparing the figures. Y.S. operated on patients and interpreted the results. All authors reviewed and revised the manuscript for intellectual content.

## Funding

The study was funded by the Beijing Natural Science Foundation‐Haidian Original Innovation Joint Fund Project (L222022); Beijing Hospitals Authority Clinical Medicine Development of Special Funding Support (ZLRK202319); and the National Key R&D Program of China (2022YFC2405302).

## Conflicts of Interest

The authors declare no conflicts of interest.

## Ethics Statement

This study was approved by the Ethics Committee of the Xuanwu Hospital, Capital Medical University.

## Consent

All participants signed written informed consent.

## Data Availability

The data that support the findings of this study are available on request from the corresponding author. The data are not publicly available due to privacy or ethical restrictions.
